# RESTING HEART RATE MODERATES THE RELATIONSHIP BETWEEN NEUROPSYCHIATRIC SYMPTOMS, MCI, AND ALZHEIMER’S DISEASE

**DOI:** 10.1093/geroni/igz038.2385

**Published:** 2019-11-08

**Authors:** Shanna L Burke

**Affiliations:** Florida International University, Miami, Florida, United States

## Abstract

Little is known about how resting heart rate moderates the relationship between neuropsychiatric symptoms and cognitive status. This study examined the relative risk of NPS on increasingly severe cognitive statuses and examined the extent to which resting heart rate moderates this relationship. A secondary analysis of the National Alzheimer’s Coordinating Center Uniform Data Set was undertaken, using observations from participants with normal cognition at baseline (13,470). The relative risk of diagnosis with a more severe cognitive status at a future visit was examined using log-binomial regression for each neuropsychiatric symptom. The moderating effect of resting heart rate among those who are later diagnosed with mild cognitive impairment (MCI) or Alzheimer’s disease (AD) was assessed. Delusions, hallucinations, agitation, depression, anxiety, elation, apathy, disinhibition, irritability, motor disturbance, nighttime behaviors, and appetite disturbance were all significantly associated (p<.001) with an increased risk of AD, and a reduced risk of MCI. Resting heart rate increased the risk of AD but reduced the relative risk of MCI. Depression significantly interacted with resting heart rate to increase the relative risk of MCI (RR: 1.07 (95% CI: 1.00-1.01), p<.001), but not AD. Neuropsychiatric symptoms increase the relative risk of AD but not MCI, which may mean that the deleterious effect of NPS is delayed until later and more severe stages of the disease course. Resting heart rate increases the relative risk of MCI among those with depression. Practitioners considering early intervention in neuropsychiatric symptomology may consider the downstream benefits of treatment considering the long-term effects of NPS.

